# The (un)suitability of modern liquid crystal displays (LCDs) for vision research

**DOI:** 10.3389/fpsyg.2015.00303

**Published:** 2015-03-23

**Authors:** Masoud Ghodrati, Adam P. Morris, Nicholas Seow Chiang Price

**Affiliations:** Department of Physiology, Monash UniversityMelbourne, VIC, Australia

**Keywords:** vision, monitor, cathode ray tube, liquid crystal display, CRT, LCD

## Abstract

Psychophysical and physiological studies of vision have traditionally used cathode ray tube (CRT) monitors to present stimuli. These monitors are no longer easily available, and liquid crystal display (LCD) technology is continually improving; therefore, we characterized a number of LCD monitors to determine if newer models are suitable replacements for CRTs in the laboratory. We compared the spatial and temporal characteristics of a CRT with five LCDs, including monitors designed with vision science in mind (ViewPixx and Display++), “prosumer” gaming monitors, and a consumer-grade LCD. All monitors had sufficient contrast, luminance range and reliability to support basic vision experiments with static images. However, the luminance of all LCDs depended strongly on viewing angle, which in combination with the poor spatial uniformity of all monitors except the VPixx, caused up to 80% drops in effective luminance in the periphery during central fixation. Further, all monitors showed significant spatial dependence, as the luminance of one area was modulated by the luminance of other areas. These spatial imperfections are most pronounced for experiments that use large or peripheral visual stimuli. In the temporal domain, the gaming LCDs were unable to generate reliable luminance patterns; one was unable to reach the requested luminance within a single frame whereas in the other the luminance of one frame affected the luminance of the next frame. The VPixx and Display++ were less affected by these problems, and had good temporal properties provided stimuli were presented for 2 or more frames. Of the consumer-grade and gaming displays tested, and if problems with spatial uniformity are taken into account, the Eizo FG2421 is the most suitable alternative to CRTs. The specialized ViewPixx performed best among all the tested LCDs, followed closely by the Display++; both are good replacements for a CRT, provided their spatial imperfections are considered.

## Introduction

Vision science experiments have historically depended upon cathode-ray tube (CRT) monitors to present stimuli with high spatial and temporal acuity. However, due to competition from plasma screens and liquid crystal displays (LCDs), CRT production was reduced or ceased by most manufacturers throughout the mid-2000s, meaning that many vision scientists now largely depend upon old and increasingly unreliable CRT monitors. Although CRTs are far from perfect numerous studies have described their superior performance relative to LCDs (e.g., Elze et al., 2007; Elze and Tanner, 2011, 2012). In recent years, however, high-quality LCDs targeting gamers have become commercially available. Further, two specialized companies now produce LCDs that aim specifically to meet the needs of vision researchers (“ViewPixx,” VPixx Technologies Inc., Canada, and “Display++,” Cambridge Research Systems, UK). Here, we compare the spatial and temporal luminance characteristics of these high-end research LCD monitors with more readily available gaming LCDs and CRTs.

Although CRTs are often considered the gold-standard monitor for use in vision research, they still have a number of limiting features (García-Pérez and Peli, 2001). CRTs generate an image by focussing an electron beam onto a phosphor layer, which emits visible light when struck by an electron. Color monitors use three phosphor layers, which each emit light with a different wavelength. The electron beam is rapidly “raster” scanned in rows, from the top-left to the bottom-right of the monitor, meaning that the entire image cannot be updated simultaneously. The decay rate of the phosphor's fluorescence, in combination with the rapid electron scanning, means that CRT monitors are unable to deliver continuous luminance patterns and the emitted light flickers at the frame rate used to generate images. This flicker is typically at 60–120 Hz, and while typically perceptually invisible, can strongly affect neural responses to visual stimuli (Wollman and Palmer, 1995; Krolak-Salmon et al., 2003; Williams et al., 2004). The relatively poor spatial independence in adjacent pixels in CRT monitors is also a major drawback, introducing artifacts to stimuli with high spatial frequency (Cowan, 1995; Bach et al., 1997; Pelli, 1997a; Krantz, 2000). Some older CRTs exhibit problems with focus of the electron beam and unreliable reproduction of low luminances.

LCDs use liquid crystals as voltage-controlled filters to control light emission. Light from a source at the back of the monitor (e.g., a light emitting diode or cold cathode fluorescent lamp) passes through three consecutive filtering layers: a polarizing filter; a layer of liquid crystals; and finally a second polarizing filter oriented orthogonal to the first. Light intensity is determined by the level of polarization change introduced by the liquid crystal layer: if no voltage is applied to the liquid crystals, they align such that the liquid crystal layer introduces a 90° change in polarization angle, and maximum light intensity will be achieved. As the voltage applied to the liquid crystals increases, they progressively change alignment, blocking more light. Unlike CRTs, each pixel in an LCD monitor is an independent filter element, allowing independent adjustment of the luminance of each pixel (Krantz, 2000; Wang and Nikolic, 2011). Nevertheless, LCD displays do exhibit two key temporal problems: first, temporal artifacts may arise depending on whether the light source is continuously on, flashed briefly once per frame, or subjected to pulse width modulation (PWM) to control brightness (Elze et al., 2007; Liang and Badano, 2007; Elze and Tanner, 2011, 2012). Second, significant temporal constraints occur due to the sluggish nature of switching in liquid crystals. This latency is undesirable in experiments with rapidly changing or fast moving stimuli, as the slow dynamics causes problems such as motion blur (Hong et al., 2005; Pan et al., 2005; Someya and Sugiura, 2007; Becker, 2008; Feng et al., 2008; Watson, 2010). Furthermore, the measured light intensity can dramatically change as the visual angle of the observer varies.

The demands of vision scientists using moving or reverse correlation stimuli, which are updated rapidly, are similar to the demands of many computer games. These games have driven the development of LCD monitors with high spatial resolution, high temporal refresh rates (100–120 Hz) and precise control over when and where light is emitted from the monitor on each frame refresh. Recent advancements in display technology have also facilitated the development of professional LCD monitors that are intended to meet the spatial and temporal requirements of vision science experiments. Given the constraints of CRT monitors, and the rapidly expanding market for LCD monitors, we examined whether any LCD monitors are suitable replacements for CRTs in the laboratory. In this study, we characterized and compared the spatial and temporal properties of a CRT monitor, two LCD monitors made specifically for vision sciences, two high quality gaming LCDs, and a consumer-grade LCD.

Our spatial tests demonstrate that most LCDs exhibit a dramatic decline in luminance toward their periphery, with effective luminance also dependent on viewing angle. The Display++ and VPixx are less strongly affected by this peripheral decline in luminance, and provide hardware based methods that partially compensate for luminance anisotropies in the vertical axis. All consumer-level and gaming LCDs showed difficulties in generating reliable temporal precision: (1) they were unable to reach the requested luminance within a single frame; and (2) they showed temporal dependence, meaning that the luminance of one frame affects the subsequent frame. The Display++ and VPixx were better in this regard, but care needs to be taken when calculating the actual duration of single-frame stimuli as the hardware mechanisms that control light generation and light transmission cannot be perfectly synchronized. We emphasize that depending on the type of experiments, extreme caution should be taken in selecting any LCD monitor to replace a CRT; however, we are confidently using the vision-science specific LCDs for electrophysiological and psychophysical studies.

## Materials and methods

### Experimental setup

We measured the temporal and spatial luminance characteristics of one cathode ray tube (CRT; Sony CPD-G520) and five liquid crystal display (LCD) monitors (Table 1). The CRT was purchased new in 2001 and has been used for occasional vision-science testing since that time. All electron guns are functional and focused. Our test set included LCDs made specifically for vision science experiments (VPixx ViewPixx 3D Lite and Cambridge Research Systems Display++), high-end gaming monitors (Samsung 2232RZ and EIZO FG2421), and a consumer-grade monitor (Dell 2209WA). All monitors were purchased by the authors, and the authors have no affiliation or association with any of the manufacturers.

**Table 1:** **Monitor specifications and basic luminance characteristics**.

**Monitor**	**Refresh rate (Hz)**	**Resolution (pixel)**	**Max. Luminance^#^ (cd/m^2^)**	**Min. Luminance (cd/m^2^)**	**Contrast (%)**	**Viewable area (mm)**
EIZO FG2421 LCD (Turbo 240 mode)	120	1920 × 1080	158.4 ± 0.14	0.02 ± 0.01	100.0	295 × 515
VIEWPixx 3D Lite LCD (Scrolling mode)	120	1920 × 1080	106.6 ± 0.09	0.25 ± 0.03	99.5	295 × 520
Samsung 2232RZ LCD	120	1680 × 1050	225.6 ± 0.40	1.84 ± 0.04	98.4	295 × 475
Dell 2209WA LCD	60	1680 × 1050	193.8 ± 0.26	1.45 ± 0.05	98.5	295 × 475
CRS Display++ LCD (Scrolling mode)	120	1920 × 1080	234.0 ± 1.85	0.40 ± 0.02	99.7	395 × 705
Sony CPD-G520 CRT	85	1280 × 1024	54.4 ± 0.39	0.04 ± 0.03	99.9	305 × 405

# Luminance values are the mean ± SD of 6 measurements. Maximum luminance under our test conditions. All monitors had methods for scaling the luminance, so the maximum achievable luminance for each monitor is higher than reported.

Visual stimuli were generated using an NVIDIA GeForce GTX 650 graphic card (memory 4 GB; 8 bits color resolution) under the control of MATLAB with the Psychophysics Toolbox (Brainard, 1997; Pelli, 1997b) in 32-bit Microsoft Windows. Luminance measurements were made in three ways: (1) using a computer-controlled spectrophotometer placed flush against the screen to block any stray light (i1, X-Rite); (2) a monolithic photodiode placed on the screen (Opt101, Burr-Brown Products, Texas Instruments); and (3) a spot-photometer with 1/3° acceptance angle (LS-110, Konica Minolta).

The spectrophotometer returned luminance measurements in CIE Lxy color space as well as raw spectral data between 380 and 730 nm at 10 nm intervals. Prior to experiments, the sensor was calibrated using a standard white calibration plate.

The photodiode voltage output was monotonically related to luminance and was used for temporal characterization of each monitor. The output was amplified and sampled at 30 KHz (Cereplex Direct, Blackrock Microsystems).

The spot-photometer was mounted on a tripod 1 m from the screen and was used to measure the luminance at the center of each monitor at viewing angles spanning 0–45° azimuth, 0–30° elevation.

The default settings of monitors for brightness, contrast, and color were used for all measurements, with no gamma correction. We also used the native display resolution (Table 1) for each monitor during the measurements. All tests were performed in a dark room with no other sources of light. All luminance stimuli were presented to the full screen, apart from the test of spatial independence.

### Experiments

#### Luminance variability

The most basic requirement of an experimental monitor is that it reliably generates a range of luminance levels. To assess luminance variability, we presented (nominally) uniform, full screen images with 32 gray levels from black (i.e., RGB = 0, 0, 0) to white (i.e., RGB = 255, 255, 255). Each image was presented for 1 s and spectral data was collected using the X-Rite i1Pro positioned at the center of the screen, beginning 50 ms after the luminance change. Gray-level was sequentially increased, with each luminance ramp (32 gray levels) repeated six times. The CRT was turned on for 30 min before testing, minimizing variability due to warming up (Klein et al., 2013).

#### Spatial variability in luminance

An ideal vision science monitor must be spatially uniform; that is, the luminance produced for a given input intensity should be the same at all positions on the screen. To characterize spatial inhomogeneities in luminance, we repeated the assessment of luminance variability describe above but made measurements at nine equally spaced positions and with only three stimulus levels (black, mid-gray and white). The nine positions were chosen as the centers of a 3 × 3 uniform rectangular grid tiling the monitor.

#### Luminance dependence on viewing angle

In many vision science experiments, viewing distances of 300–570 mm are used, meaning that the projection angle of a pixel to the retina changes substantially across the monitor. For example, at the commonly used viewing distance of 570 mm, the Display++ (which spans 395 × 705 mm), the images span ~38° × 64°. For an ideal monitor, the incident luminance should not depend on viewing angle. We measured the luminance at the center of the screen with a spot photometer under three sets of conditions: (1) four azimuth angles (0°, 15°, 30°, and 45°) with 0° elevation; (2) three elevation angles (0°, 15°, and 30°) with azimuth 0°; (3) three elevation angles (0°, 15°, and 30°) with azimuth 45°. Note that the distance of the photometer from the monitors was fixed at 1 meter in all conditions.

#### Luminance spatial dependence

Ideally, changing the luminance in one part of the screen should not affect the luminance in other parts. Although LCDs allow the luminance of each pixel to be independently defined, we examined how changes in luminance across a large number of pixels affected distant parts of the monitor. We measured the luminance of a 400 × 400 pixels image with constant mid-gray (RGB = 128, 128, 128) at the upper-left corner of the monitor (using the X-Rite i1Pro) while the rest of the monitor was changed between black and white every second.

#### Temporal response to luminance alteration

To test the temporal precision of each monitor, we presented full screen white images (i.e., maximum intensity) for one or two frames, with an intervening black screen (i.e., minimum intensity) for 1 s. Each flash duration was repeated 100 times and the temporal changes in luminance were measured using an analog photodiode placed at the upper-left corner of the monitor, sampled at 30 KHz. We focused on testing the timing of black-to-white transitions, as these are commonly used in our electrophysiological and psychophysical testing. Note that black-to-white transitions are not necessarily slowest, as gray-to-gray transitions can take longer in some situations (Elze and Tanner, 2012).

## Results

The spatial and temporal specifications of each monitor are summarized in Table 1, along with the maximum and minimum luminance measured in the center of the monitor. All monitors allow an acceptable range of contrasts (>98%); however, the Dell and Samsung monitors may be inappropriate in some situations as their minimum luminance exceeded 1 cd/m^2^, leading to minimum contrasts of 98.4% and 98.5%, respectively.

**Figure 1:**
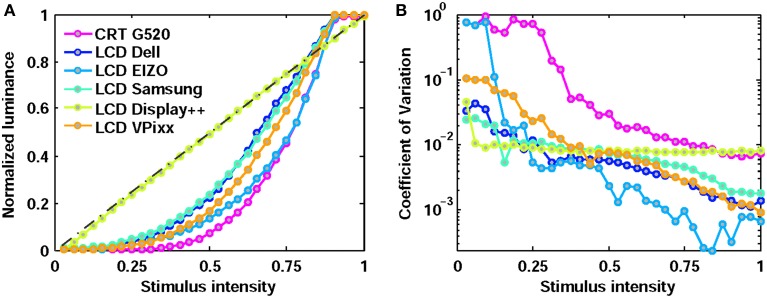
**Normalized luminance output (A) and variability (coefficient of variation; B) across 32 gray levels of input stimulus for six monitors**. The Display++ incorporates manufacturer-implemented gamma correction, providing linearized output. All other monitors were tested in their default mode, without gamma correction.

Ideally, monitors should generate constant luminance output for a given input intensity across stimulus repetitions. We examined the variability in each monitor's luminance output across a range of stimulus gray levels. The average normalized luminance and coefficient of variation for 32 equally spaced gray levels are shown in Figure 1. All luminances are normalized relative to their maximum value (Table 1).

All monitors were highly reliable (i.e., low coefficient of variation) in generating high luminances, but became more variable at lower luminances. This variability was essentially absent in the Display++, but was a marked problem for the CRT. The variability in the EIZO, evident at low luminance, is unlikely to be a practical problem as the EIZO had the darkest “black” and therefore the range of absolute luminances at this low intensity remains very small.

### Spatial characteristics

**Figure 2:**
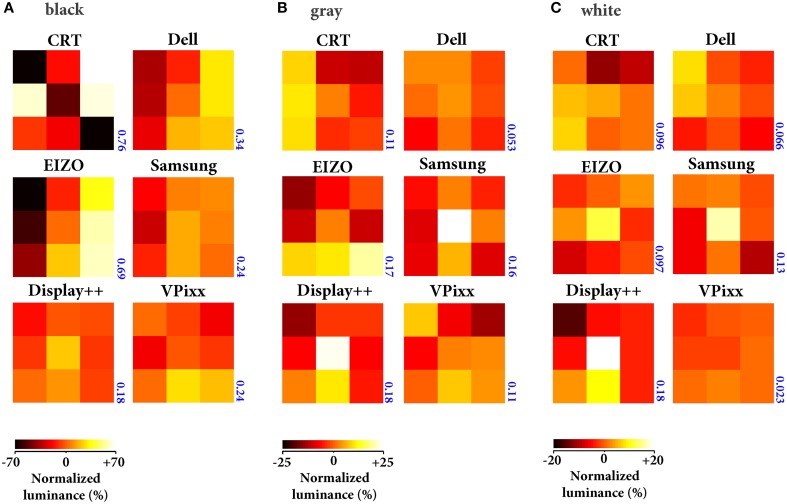
**Uniformity of spatial luminance**. Mean luminance (*n* = 6) across 9 monitor positions measured with the screen showing black **(A)**, mid-gray **(B)** and white **(C)**. Note that the heatmaps in each panel have different scaling. All luminances are expressed as a percentage of the mean across all spatial positions for a given monitor (i.e., 0 = mean luminance). The blue number next to each panel indicates the Michelson contrast for each gray level.

First, we measured the luminance in 9 equally spaced positions across three stimulus levels (black, mid-gray, and white) for full screen images. Figure 2 shows luminances normalized relative to the mean luminance recorded across all positions for a given input intensity. Surprisingly, even with a black stimulus the luminance pattern was not spatially uniform in three monitors (CRT, Dell, and EIZO). With gray and white stimuli, the EIZO, Samsung and Display++ showed up to ±20% variation in luminance across positions, with the center of the monitor markedly brighter than surrounding regions. The CRT also showed luminance variations, but this may be partly attributable to the age of the monitor (manufacture date, March 2001) and was primarily evident at extremely low mean luminances (0.04 ± 0.03 cd/m^2^) suggesting it will have no practicable effect on perception. The Michelson contrast for each gray level is indicated for each monitor, and was just 0.023 for the VPixx when presenting white, suggesting that it is effectively spatially-invariant.

**Figure 3:**
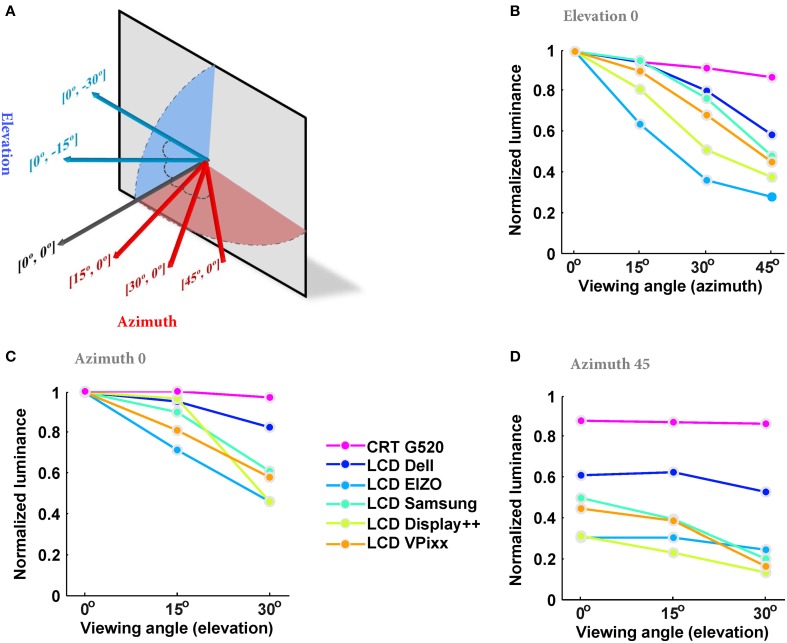
**The dependence of measured luminance on viewing angle. (A)** Different azimuth and elevation angles. The first value in the brackets indicates the azimuth and the second indicates the elevation. Each arrow shows the position and the angle of spot-photometer. **(B)** Normalized luminance at azimuth angles of 0–45° with elevation 0°. The results are the average of 4 measurements, normalized to the maximum luminance of each monitor measured with azimuth 0° and elevation 0°. **(C)** Normalized luminance at elevations of 0–30° with azimuth 0°. **(D)** Normalized luminance at elevations of 0–30° with azimuth 45°.

Next, we examined a different kind of spatial uniformity by measuring the luminance at the center of the monitor from different viewing angles (Figure 3A). The values in the figure have been normalized relative to that measured at azimuth 0°, elevation 0°. While the CRT was nearly view-invariant, the measured luminance of all LCDs depended strongly on viewing angle. As examples, at azimuth 45° and elevation 0°, the measured luminance ranged from 50 to 80% of that measured with a frontal viewing angle [0°, 0°], and with viewing angle [45°, 30°] the measured luminance with three monitors was less than 30% of that with a frontal viewing angle.

**Figure 4:**
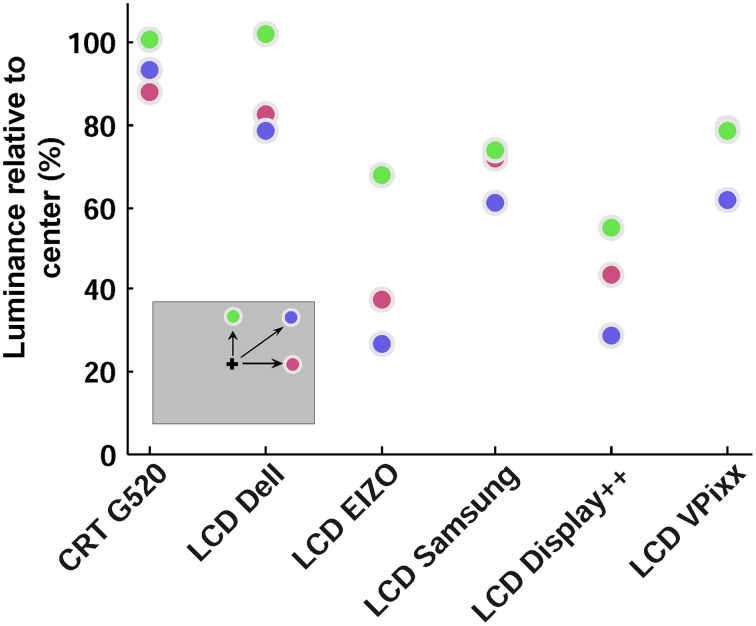
**Effect of spatial luminance variations combined with viewing angle**. Each colored circle shows relative luminance at a peripheral screen location, assuming a viewing distance of 570 mm. Positions are indicated in the representation of the monitor (inset), and were one-sixth of the monitor width and height from the right and top edges.

The combination of LCDs having brighter centers than surrounds, and luminance falling off with increasing viewing angle means that spatial anisotropy needs to be taken into account when large, or peripheral stimuli are presented. Figure 4 simulates the combined effect of using a monitor with a brighter center (Figure 2) and oblique viewing angles (Figure 3) for three peripheral positions and angles. A viewing distance of 570 mm is assumed here, however, the influence of viewing angle will increase as viewing distance decreases. To this end, we calculated the viewing angles of three points: (1) on the horizontal meridian of the monitor, one-sixth of the monitor's width from the monitor edge; (2) on the monitor's vertical meridian, one-sixth of the height from the top; and (3) one-sixth of both the width and height from the monitor edges. Then, the (normalized) luminance expected at each point for an observer directly in front of the center of the monitor was estimated by multiplying the effects of viewing angle and spatial anisotropy across the monitor surface.

By this measure, the CRT performed well, with minimal effective luminance drop from the center to the three peripheral regions. The effective luminance of all LCDs, however, decreased substantially in the periphery, with stimuli in the upper-right corner of the screen having as little as 25% of the luminance at the center of the monitor (blue circles in Figure 4). As discussed below, the VPixx and Display++ have hardware based mechanisms that can compensate for vertical luminance anisotropy by using a brighter backlight at the top and bottom of the monitor. This mechanism cannot compensate for horizontal anisotropies as the LEDs are arranged along the side of the monitor and illuminate entire rows of pixels.

**Figure 5:**
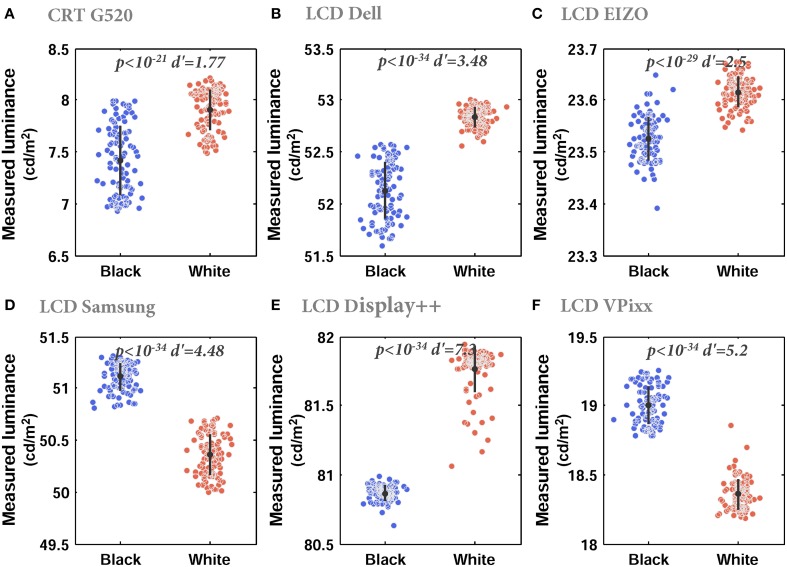
**Luminance spatial dependence**. **(A–F)** Measured luminance of a gray image when the remainder of the monitor was black or white for CRT **(A)**, Dell **(B)**. Each dot indicates an independent luminance measurement. Error bars show the mean and standard deviation. Mean luminances in the black and white conditions were significantly different for all monitors (Wilcoxon rank sum test, *p*-values at the top of each plot), with large statistical effect sizes (d′) but small differences in absolute terms.

For the final test of spatial characteristics, we measured the spatial dependence of luminance, i.e., the extent to which modulation of one part of the screen affected other parts of the screen. For all monitors, the luminance of a gray region was significantly affected by the luminance of adjacent regions (Figure 5), with the *smallest* effect size still *d* = 1.77 (where Cohen's d is the difference between the two means divided by the pooled standard deviation). Surprisingly, the directionality of this effect was variable: the luminance of the gray region was significantly higher (brighter) when other parts of the monitor were white vs. black in four monitors, and significantly lower (dimmer) when surrounded by white vs. black in two monitors. Fortunately, the absolute variations in luminance were typically less than 1 cd/m.

### Temporal dynamics

**Figure 6:**
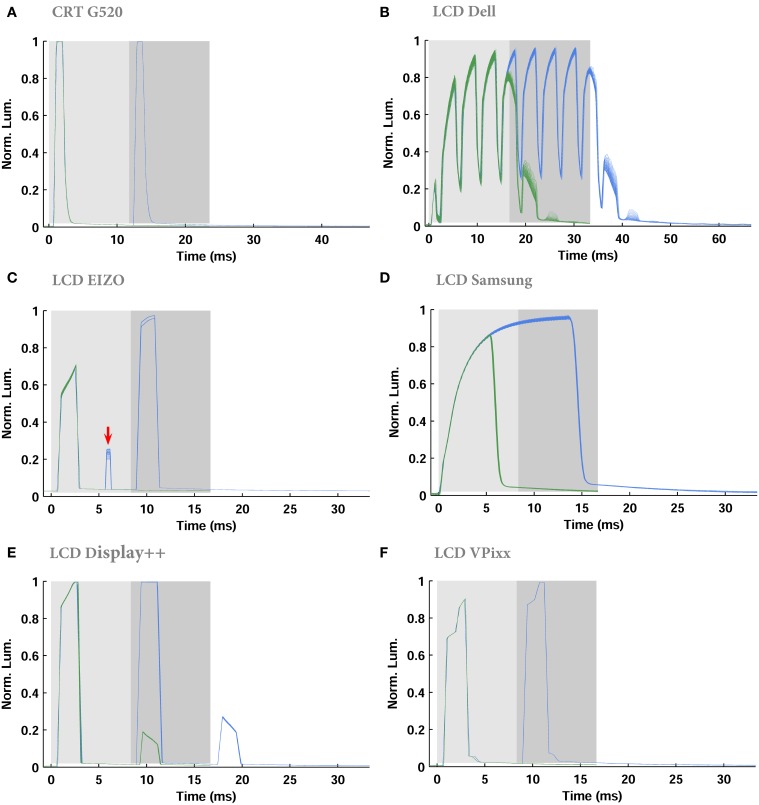
**Temporal profile of luminance “on” and “off” luminance steps**. **(A–F)** Each plot shows the temporal changes during 1 (green) and 2 (blue) frames of white stimuli over 100 repetitions, overlaid on each other. Each frame of white stimulus is indicated by the shaded regions (light gray: first frame, dark gray: second frame). The red arrow **(C)** highlights a luminance bump due to the use of Turbo-240 mode, which effectively runs the monitor at 240 Hz, with every second frame displaying black.

An ideal monitor should: (1) reliably reproduce a temporal luminance profile across multiple repetitions; (2) instantly and accurately update the luminance at any position on the monitor; and (3) generate a luminance that is independent of previously presented luminances. Figure 6 shows the temporal dynamics of luminance changes for 100 repetitions of a stimulus comprising a white screen presented for either one or two frames, following a 1 s period of black. White stimuli presented for three or more frames were not noticeably different from two-frame stimuli. The CRT represents the “gold standard”: the luminance profile within a frame rises rapidly to a peak; this peak luminance is independent of the luminance in earlier frames; and the luminance profile is reliable across stimulus repetitions (Figure 6A). All LCD monitors deviated from the benchmark, with the VPixx showing the best overall performance.

For all LCD monitors except the Dell, there was little noticeable variability in the temporal luminance profile, highlighted by the fact that the overlayed traces from 100 stimulus trials are almost indistinguishable (Figures 6B–F). The Dell monitor was highly variable across stimulus repetitions and flickered in luminance even within a single frame.

All LCDs showed some level of luminance hysteresis when stimuli were changed from black to white, or white to black (Figure 6). Starting from a black screen, all LCDs were unable to reach their maximum luminance in a single frame. Due to the range of backlight mechanisms implemented in the monitors, we have not attempted to quantify the rate of increase or decrease in luminance. The Display++ and VPixx, however, had the best performance as the luminance: (1) rose to close to its peak for any given frame within <2 ms; and (2) rose close to the peak possible luminance on the first frame of a white stimulus following black. Note that the step-like luminance transitions evident with these monitors reflect the progressive illumination of different LEDs associated with the scrolling backlight mode. After two frames of white, the Dell and Display++ were the only LCDs unable to switch back to black within a single frame. This is evident as the non-zero luminance bumps after the gray-shaded regions in Figures 6B,E (from 35 to 40 ms for the Dell and 18–20 ms for the Display++). For these monitors, the first black frame following a white frame would appear as gray. Note that in the case of the Display++, this extra flash appeared regardless of whether the monitor was run in strobing or scrolling backlight mode. Collectively, the hysteresis effects cause two problems: briefly presented luminance increments (e.g., black-white-black) last one frame longer than desired, and transitions between extreme stimulus levels (e.g., black to white) do not occur within a single frame. One observation warrants special attention—the EIZO had a luminance “bump” in the first white frame that is only evident when two or more white frames are presented (arrow in Figure 6C). This is due to the use of “Turbo 240” mode, which uses an effective backlight frequency of 240 Hz.

## Discussion

We assessed five LCD monitors in order to determine if their temporal and spatial characteristics make them suitable replacements for CRT monitors in vision research. In our spatial tests, we measured how luminance varied across repeated measurements, spatial position, spatial context (i.e., the luminance of surrounding pixels), and viewing angle. In our temporal tests, we examined how reliably each monitor could produce luminance changes across consecutive frames. We did not assess the chromatic properties of each display. Our spatial and temporal measurements confirm the known limitations of CRT and conventional LCD monitors. However, we demonstrate that under some circumstances, some high-end gaming monitors meet the temporal demands of vision research, and recently available professional LCD monitors can replace CRTs in vision laboratories for most experiments.

### Spatial characteristics

#### Basic characteristics (contrast and luminance range)

All LCD monitors had reproducible outputs, although the luminances did not always match the desired values when short duration stimuli were used. Two monitors (the Dell and Samsung) were problematic in that they had noticeable light emission (>1 cd/m^2^) even when a black stimulus was presented. This is undesirable because it limits the peak contrast of the monitor. The Display++ benefits from a built-in gamma correction mechanism, which means there is a linear relationship between the drive stimulus and the screen luminance, and luminance variability is proportional to mean luminance across all drive levels.

#### Spatial uniformity and the effect of viewing angle

All LCDs except the VPixx had poor spatial uniformity, with the center typically the brightest position on the monitor. This limitation has previously been reported for both CRT and LCD monitors (Metha et al., 1993; Bohnsack et al., 1997; Krantz, 2000; García-Pérez and Peli, 2001; Klein et al., 2013). The remarkable uniformity of the VPixx reflects that it is designed specifically for experimental use; with other monitors, the inhomogeneity must be taken into account when the aim is to present stimuli with identical luminance and contrast at different spatial locations. Although the Display++ and VPixx both have hardware-based mechanisms for correcting vertical inhomogeneities in luminance output, a more general solution would be to use software-based corrections to the image intensities passed to the video card (e.g., by parameterizing the full input-output relationship across space, analogous to traditional gamma-correction).

While CRTs have little appreciable change in luminance across a wide range of viewing angles, for all LCDs, increasing the viewing angle to more than 15° in either axis (azimuth and elevation) greatly decreased the measured luminance. This reflects a major shortcoming of LCD technology, which is also evident in modern commercial displays based on organic light emitting diodes (OLED displays) (Ito et al., 2013). Surprisingly, the cheapest Dell LCD was the least affected by viewing angle, which may make it a convenient choice for presenting static images. With small viewing distances, the effects of viewing angle combine with the drop-off in peripheral pixel luminance, such that if a subject looks at the upper-right corner of the screen from the center with viewing distance of 570 mm, the luminance fall off for LCDs is huge (almost 80% for EIZO and 40% for VPixx relative to center). This dramatic luminance drop must be taken into account when stimuli are presented in peripheral regions.

#### Luminance spatial dependence

Spatial dependence is commonly tested by comparing the spatially-averaged luminance of horizontal and vertical gratings comprising alternating black and white pixels. CRTs “fail” this test as vertical gratings have lower luminances than horizontal gratings (e.g., Pelli, 1997a; Krantz, 2000; Wang and Nikolic, 2011). LCD technology overcomes this problem as it independently addresses each pixel, making the monitor spatially independent. However, here we took another approach and measured the interactions between large regions of the monitor. In all LCDs, we observed that even slow modulations in one area of the monitor from black to white caused significant luminance changes in other regions. However, it should be noted that the absolute changes in measured luminance were very small and therefore this effect can probably be ignored.

### Temporal characteristics

The temporal characteristics of a monitor can be assessed by examining: (1) whether the requested luminance is reached within a single frame; (2) the reliability of temporal luminance patterns across multiple repetitions; (3) the independence of luminance in consecutive frames; (4) the rise and fall times associated with luminance changes within a frame. We measured the first three of these characteristics, but it was inappropriate to compare rise and fall times due to the different light sources and modes of illumination in each monitor.

Our measurements show that the CRT and the VPixx satisfied all temporal factors; both provided highly reliable luminance profiles within a single frame and minimal interaction between consecutive frames. However, our results suggest that most consumer-grade and gaming LCD monitors cannot safely be used for all experiments requiring temporally-precise stimuli. For example, the Dell LCD exhibited multiple luminance alterations within a single frame (with frequency of ~240 Hz) due to the backlight controller. Luminance output for this monitor was also highly variable across repetitions, did not reach the desired maximum luminance even after the second frame, and was very slow at falling to baseline luminance at the end of a frame.

The Samsung LCD has been described previously as a monitor suitable for vision science (Wang and Nikolic, 2011), and we found it to be highly reliable and temporally precise. However, as it has continuous light emission, it implements “sample and hold” style stimuli, which will lead to blur artifacts when using moving stimuli. Thus, the Samsung is appropriate for stimuli requiring high temporal precision such as flashes and reverse correlation, but not motion.

The Display++ and EIZO LCD exhibited interactions between the luminance of consecutive frames. Although this interaction may have minimal perceptual effect, it cannot be easily neglected in physiology experiments, as it would likely be detected by neurons early in the visual processing hierarchy (De Lange Dzn, 1954; Smith et al., 2001). A number of factors could ameliorate this problem. First, in our tests, we used stimuli that changed from minimum to maximum intensity, which require the largest state change in the liquid crystals. Using lower contrast changes (e.g., 10% or 50%–90% luminance) should allow faster switching. Second, given that our results suggest that the timing of these monitors is highly reliable, their imperfections could be incorporated into most experimental designs. Finally, the timing and intensity of the backlight in the Display++ and VPixx is user-configurable. The backlight comprises 32 LEDs along the left side of each monitor, which can be strobed simultaneously or illuminated sequentially in a scrolling manner. Illuminating a given LED as late as possible after the corresponding row of pixels has been updated is the best way to minimize luminance cross-talk between frames.

### Other points and conclusion

Table 2 summarizes the strengths and weaknesses of each monitor. In our opinion, none of the consumer-level or gaming LCDs can safely be used as a replacement for a CRT in *all* vision science experiments. Provided motion stimuli are not required, the Samsung provides excellent temporal fidelity and continuous light output. Provided the luminance-interaction between frames is incorporated into stimulus design, the Eizo also provides excellent temporal fidelity. We would not recommend either the Samsung or Eizo for stimuli requiring spatial uniformity of luminance. As the specifications of prosumer gaming monitors continually changes, it is critical that they are field-tested before being used in experimentation. We strongly recommend that if stimulus timing is important, users test the temporal fidelity of even gaming-level monitors. This is cost-effectively achieved by placing a phototransistor or light-dependent resistor in front of the monitor and measuring the light output associated with the desired stimulus. The output of the photosensor can be visualized using an oscilloscope (PC-based oscilloscopes are available for ~$150USD). In most cases, the photosensor output will require amplification, but in some cases, even this is unnecessary. The magnitude of the photosensor output is not important here, only its timing. Discrete luminance peaks associated with each frame refresh should be evident (e.g., Figure 6), and the number of peaks must match the number of frames in the desired stimulus.

**Table 2:** **Summary of the strengths and weaknesses of each monitor**.

**Monitor**	**Strengths**	**Weaknesses**	**Appropriate for**
VIEWPixx 3D Lite	Scrolling backlight and high refresh rate. Temporally precise.	Luminance variations with viewing angle and position	All testing scenarios, provided peripheral luminance variations are considered
CRS Display++	Scrolling backlight and high refresh rate	Temporal interactions between frames	All testing scenarios, provided peripheral luminance variations and temporal characteristics are considered
EIZO FG2421	High refresh rate (with 240 Hz mode)	Luminance variations with viewing angle and position	All testing scenarios, provided peripheral luminance variations and temporal characteristics are considered
Samsung 2232RZ	High refresh rate and temporal precision	Continuous light output	Static images requiring good temporal precision
Dell 2209WA	Lowest luminance variations with viewing angle and position	Poor temporal resolution	Static images, where temporal precision is not required

Monitors are ordered in terms of their general suitability for vision research.

Finally, the VPixx, and to a lesser degree, the Display++ are excellent replacements for CRTs. Although not described above, in addition to their temporal and spatial precision, the VPixx and Display++ are compatible with MATLAB and PsychToolbox, which are widely used in vision sciences. Both monitors incorporate 10-bit RGB, digital inputs and outputs, optional analog I/O and can incorporate optional touchscreens. All I/O timing is synchronized with that of monitor refreshes. Thus, these modern monitors provide significant benefits in terms of ease of experimental control compared to traditional CRTs and we are confident in using them for psychophysical, oculomotor and physiological studies.

### Conflict of interest statement

The authors declare that the research was conducted in the absence of any commercial or financial relationships that could be construed as a potential conflict of interest.

## References

[B1] Bach M. Meigen T. Strasburger H. (1997). Raster-scan cathode-ray tubes for vision research-limits of resolution in space, time and intensity, and some solutions. Spat. Vis. 10, 403–414. 10.1163/156856897X00311 9176948

[B2] Becker M. E. (2008). Motion-blur evaluation: a comparison of approaches. J. Soc. Inf. Disp. 16, 989–1000. 10.1889/JSID16.10.98918044609

[B3] Bohnsack D. L. Diller L. C. Yeh T. Jenness J. W. Troy J. B. (1997). Characteristics of the Sony Multiscan 17se Trinitron color graphic display. Spat. Vis. 10, 345–351. 10.1163/156856897X00267 9176943

[B4] Brainard D. H. (1997). The psychophysics toolbox. Spat. Vis. 10, 433–436. 10.1163/156856897X00357 9176952

[B6] Cowan W. B. (1995). Displays for vision research. Handb. Opt. 1, 27–21.

[B7] De Lange Dzn H. (1954). Relationship between critical flicker-frequency and a set of low-frequency characteristics of the eye. J. Opt. Soc. Am. 44, 380–388. 10.1364/JOSA.44.000380 13163770

[B8] Elze T. Lochmann T. Tanner T. G. (2007). Does flat mean slow? LCD monitors and their temporal precision for visual experiments, in 30th European Conference on Visual Perception (Arezzo), 162.

[B9] Elze T. Tanner T. (2011). Dangerous liquids: temporal properties of modern LCD monitors and implications for vision science experiments, in 34th European Conference on Visual Perception (Toulouse), 175.

[B10] Elze T. Tanner T. G. (2012). Temporal properties of liquid crystal displays: implications for vision science experiments. PLoS ONE 7:e44048. 10.1371/journal.pone.0044048 22984458 PMC3439495

[B11] Feng X. Pan H. Daly S. (2008). Comparisons of motion-blur assessment strategies for newly emergent LCD and backlight driving technologies. J. Soc. Inf. Disp. 16, 981–988 10.1889/JSID16.10.981

[B12] García-Pérez M. A. Peli E. (2001). Luminance artifacts of cathode-ray tube displays for vision research. Spat. Vis. 14, 201–215. 10.1163/156856801300202931 11450803

[B13] Hong S. Berkeley B. Kim S. S. (2005). Motion image enhancement of LCDs, in IEEE International Conference on Image Processing, 2005. ICIP 2005 (Genova: IEEE), II–17.

[B14] Ito H. Ogawa M. Sunaga S. (2013). Evaluation of an organic light-emitting diode display for precise visual stimulation. J. Vis. 13:6. 10.1167/13.7.6 23757510

[B15] Klein J. Zlatkova M. Lauritzen J. Pierscionek B. (2013). Photometric and colorimetric measurements of CRT and TFT monitors for vision research. J. Mod. Opt. 60, 1159–1166 10.1080/09500340.2013.808385

[B16] Krantz J. H. (2000). Tell me, what did you see? The stimulus on computers. Behav. Res. Methods Instrum. Comput. 32, 221–229. 10.3758/BF03207787 10875166

[B17] Krolak-Salmon P. Hénaff M.-A. Tallon-Baudry C. Yvert B. Guénot M. Vighetto A.. (2003). Human lateral geniculate nucleus and visual cortex respond to screen flicker. Ann. Neurol. 53, 73–80. 10.1002/ana.10403 12509850

[B18] Liang H. Badano A. (2007). Temporal response of medical liquid crystal displaysa. Med. Phys. 34, 639–646. 10.1118/1.2428403 17388181

[B19] Metha A. B. Vingrys A. J. Badcock D. R. (1993). Calibration of a color monitor for visual psychophysics. Behav. Res. Methods Instrum. Comput. 25, 371–383. 10.3758/BF032045289176958

[B20] Pan H. Feng X.-F. Daly S. (2005). LCD motion blur modeling and analysis, in IEEE International Conference on Image Processing, 2005. ICIP 2005 (Genova: IEEE), II–21.

[B21] Pelli D. G. (1997a). Pixel independence: measuring spatial interactions on a CRT display. Spat. Vis. 10, 443–446. 10.1163/156856897X00375 9176954

[B22] Pelli D. G. (1997b). The VideoToolbox software for visual psychophysics: transforming numbers into movies. Spat. Vis. 10, 437–442. 10.1163/156856897X00366 9176953

[B23] Smith V. C. Pokorny J. Lee B. B. Dacey D. M. (2001). Primate horizontal cell dynamics: an analysis of sensitivity regulation in the outer retina. J. Neurophysiol. 85, 545–558. 11160492 10.1152/jn.2001.85.2.545

[B24] Someya J. Sugiura H. (2007). Evaluation of liquid-crystal-display motion blur with moving-picture response time and human perception. J. Soc. Inf. Disp. 15, 79–86 10.1889/1.2451570

[B25] Wang P. Nikolic D. (2011). An LCD monitor with sufficiently precise timing for research in vision. Front. Hum. Neurosci. 5:85. 10.3389/fnhum.2011.00085 21887142 PMC3157744

[B26] Watson A. B. (2010). Display motion blur: comparison of measurement methods. J. Soc. Inf. Disp. 18, 179–190 10.1889/JSID18.2.179

[B27] Williams P. E. Mechler F. Gordon J. Shapley R. Hawken M. J. (2004). Entrainment to video displays in primary visual cortex of macaque and humans. J. Neurosci. 24, 8278–8288. 10.1523/JNEUROSCI.2716-04.2004 15385611 PMC6729686

[B28] Wollman D. E. Palmer L. A. (1995). Phase locking of neuronal responses to the vertical refresh of computer display monitors in cat lateral geniculate nucleus and striate cortex. J. Neurosci. Methods 60, 107–113. 10.1016/0165-0270(94)00226-7 8544468

